# Analysis of the Bearing Capacity of Helical Pile with Hexagonal Joints

**DOI:** 10.3390/ma11101890

**Published:** 2018-10-02

**Authors:** Daehyeon Kim, Kyemoon Baek, Kyungho Park

**Affiliations:** 1 Department of Civil Engineering, Chosun University, Gwangju 61452, Korea; dkimgeo@chosun.ac.kr; 2 SOIL-ROCK E&G CO., Ltd., Jeollabuk-do 55340, Korea; soil-rock@hanmail.net

**Keywords:** hexagonal joint, helical pile, bearing capacity, static pile load test, dynamic pile load

## Abstract

This study aims to improve shafts with hexagonal joints so that they will not require welding or bolts in static load tests. In order to evaluate the bearing capacity of helical piles, two sites were selected to conduct pile installation for the field test and the pile load test. For the pile load test, a static pile load test and a dynamic pile load test were carried out, and torque was measured during pile installation in a field test to compare and analyze the expected bearing capacity and thus assess the feasibility of the method for estimating the bearing capacity. The field pile load test revealed that the bearing capacity of the gravity grout pile was the same or greater than 600 kN in the static pile load test in accordance with the AC 358 code. The non-grout pile showed a bearing capacity that was the same or smaller than 600 kN, suggesting that gravity grouting is required. Moreover, the field pile load test was used to establish the bearing capacity equation considering the torque in the pile installation, and a small number of samples were used to establish the equation, which can be used as basic data.

## 1. Introduction

A helical pile is a non-displacement pile foundation that implements bearing capacity by attaching at least one helix plate to a hollow shaft to be rotary-penetrated into the ground. The helical pile can be installed with low-noise and low-vibration performance by means of a torque machine that guides rotary penetration to a target depth. This is a pile that can be installed with a comparatively small machine and in locations with limited installation spaces, for example, commercial buildings or historic sites. The helical pile is better than conventional steel pipe piles in terms of bearing capacity for the material costs, because helix plates with a diameter larger than the hollow shaft are attached to it to allow each helix plate to have end bearing capacity [1].

Most helical piles have a standardized shape, and the bearing capacity depending on the hollow diameter and the helix diameter have been studied. Although most helical piles are installed by means of bolts and on-site welding for simple installation, there are issues involved in poor verticality in pile installation, worker’s safety related to bolt damage in rotary penetration, on-site labor costs for welders and an increase in the installation period due to the additional welding process and further quality control of the welded locations. Therefore, it is necessary to apply a new type of joint to address the aforementioned issues.

Furthermore, because the shape of helical piles is different from that of the conventional piles, using conventional equations is not ideal in terms of high reliability, and it is necessary to examine the bearing capacity of the helical piles installed in Korea using conventional equations [2]. 

Helical piles were invented by the Irish civil engineer Alexander Mitchell in 1836 to reinforce the foundation of houses. Helical piles have been used in the UK since 1853, and were used in the US generally as a foundation for lightweight houses between 1850 and 1890. After this period, helical piles were used for purposes similar to anchors until 1985. At present, they are generally used for transmission tower foundations, foundations for small- and medium-sized buildings and roads, and slope stabilization [3].

Helical piles have been studied, with a focus on assessing their pull-out performance when used as anchors. The authors in [4] studied the behavior of the ground around the anchor by rotary penetration (rotary penetration) under ultimate loads to establish an equation for calculating the pull-out forces of the anchor, and [5] calculated the ultimate pull-out forces through the field pull-out test. 

Clemence [6] announced the best pitch ratio to allow the soil mass between the helixes to behave as one mass through the indoor test. The authors in [7] studied the pull-out resistance characteristics of single- and multi-helix anchors installed in sandy soil and clay soil, and announced an empirical equation for calculating the penetration depth of the helical anchor, the screw wing diameter, and the pull-out resistance depending on ground conditions. 

Narasimha et al. [8] studied the effect of the shaping factor in determining the helical anchor’s effect on the pull-out forces. The authors in [9] carried out a numerical analysis of the ultimate pull-out resistance of helical anchors installed in clay soil, and examined the relation between the ultimate pull-out resistance and the specifications for installing helical piles.

Recently, helical piles have been studied in terms of the behavior of their bearing capacity. The authors in [8,10] evaluated the bearing capacity of helical piles and the behavioral characteristics of helix plates in relation to the pitch ratio, and [9] recommended the cylindrical shear method for evaluating the bearing capacity of multi-helical piles.

Perko [3] established an equation for the average diameter relative to the unit weight of helical piles for the critical depth thereof, and [4] said that the scale of lateral earth pressure on the helical piles is related to the initial relative density of the ground, and calculated the lateral earth pressure coefficient.

Ghaly and Clemence [11] studied the friction occurring in the shaft of helical piles to confirm the adhesive force occurring in the shaft of helical piles in the clean coarse sandy ground, and [12] said that the shaft adhesion force must be limited although it can be used. 

Hoyt and Clemence [13] said that the relation between the rolling resistance and bearing capacity of helical piles depends on the shaft diameter, and [14] evaluated the relation between the rolling resistance and bearing capacity during pile installation using the law of the conservation of energy. 

In most of cases, a pile foundation is composed by a pile group, in which the key factors to be considered are mainly: The pile spacing, the pile-soil-pile interactions, and the stiffness of the connecting structure [15]. Ideally, the piles in a group should be spaced so that the load-bearing capacity of the group is not less than the sum of the bearing capacity of the individual piles. The minimum center-to-center pile spacing, s, is 2.5D (Diameter of pile) and, in ordinary situations, is about 3.0–3.5D [16].

Also, the lateral resistance of the pile is very important in the design process of the foundation system. There are several methods for analyzing laterally loaded piles, and the p-y method is the most used in the common practice [17].

In Korea, [18] studied the characteristics of screw anchor pile pull-out resistance, and conducted an indoor test with different geometrical features, for example, different helix plate diameters and pitches. The result shows that the pull-out resistance increases as the helix plate increases, but is constant at sizes greater than a specific size. 

Lee et al. [19] compared the field load test result with the bearing capacity using the individual bearing method, cylindrical shear method, and the torque correlation method. The comparison shows that the torque correlation method implements the highest correlation. 

Park and Kim [20] said that 1000.0 kN of bearing capacity is ensured where the helical pile is settled in the hard ground (rock), and [21] evaluated field applicability and found that the result of the individual bearing method was similar to the result of the field pile load test.

Other countries than Korea have used helical piles in various construction sites for a long time. However, the advantages of helical piles are not well-known in Korea; there are prior studies on screw anchor piles similar to the shape of helical piles [18]; and another prior comparison and analysis of the bearing capacity of installed helical piles compared to the equation for estimating bearing capacity [22].

Therefore, in this study, a hollow shaft model is rolled with a hexagon joints to improve it as a fit type not requiring welding or bolts. Two sites were selected to apply the pile to the Korean ground and conduct piling for the field test and the load test. During the field pile load test, the result of the static pile load test and the dynamic pile load test were compared with the bearing capacity estimated by measuring the torque in the pile installation process to examine the feasibility of the method for estimating the bearing capacity.

## 2. Bearing Capacity Theory and Design of the Helical Pile 

### 2.1. Bearing Capacity of the Helical Pile

A helical pile was shaped to have helix plates attached to the hollow shaft with a diameter greater than the hollow shaft, and respective helix plates implementing end bearing capacity in addition to the skin friction capacity of the shaft to achieve the bearing capacity. The exemplary applicable methods for calculating the bearing capacity of helical piles include the individual bearing method, cylindrical shear method, and the torque correlation method. Moreover, the torque correlation method determines the bearing capacity coefficient (kt) through the force of rotary penetration into the ground during installation, and is a method for calculating the ultimate bearing capacity.

**Figure 1:**
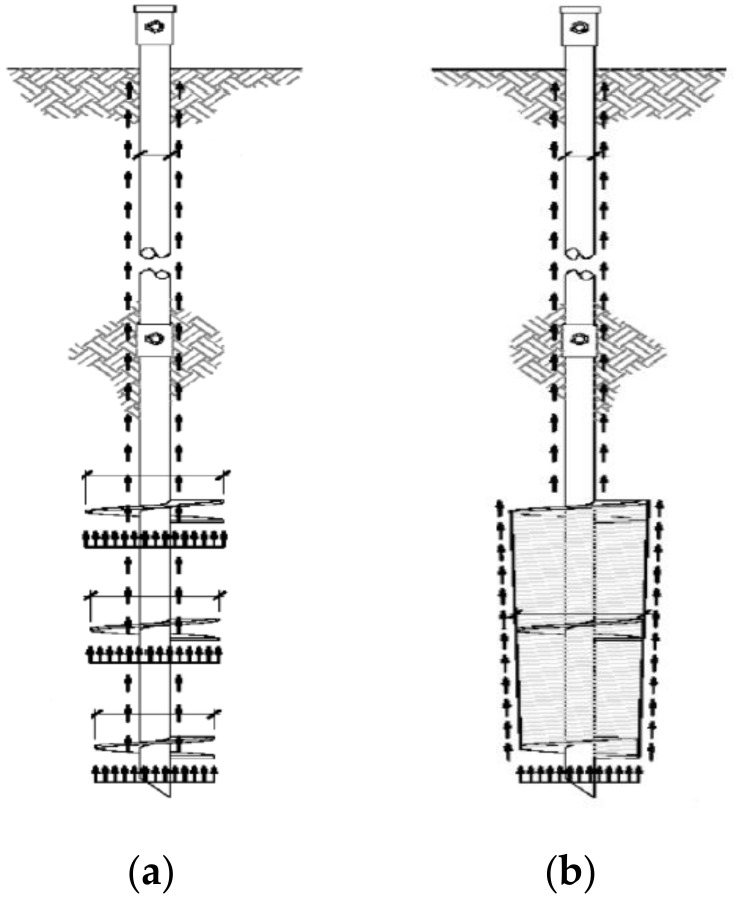
Schematic description of (**a**) Individual bearing method; (**b**) Cylindrical shear method (Perko [3]).

Figure 1 shows the bearing capacity of helical piles based on the individual bearing method and the cylindrical shear method schemes [3].

The bearing performance of helical piles is classified into end bearing capacity and skin friction capacity. In particular, the end bearing capacity is calculated using the individual bearing method and cylindrical shear method, and determined by helix spacing. Where the helix spacing is at least 2.0–3.0D_h_ of the helix plate diameter D_h_, the piles show the bearing performance of the individual bearing method. However, where they are at most 2.0–3.0D_h_ of the helix plate diameter D_h_, they show the bearing performance of the cylindrical shear method [3,23,24]. Moreover, the skin friction capacity can be calculated by means of skin friction between the upper shaft of the helix plates and the ground.

### 2.2. Details of the Helical Pile

**Figure 2:**
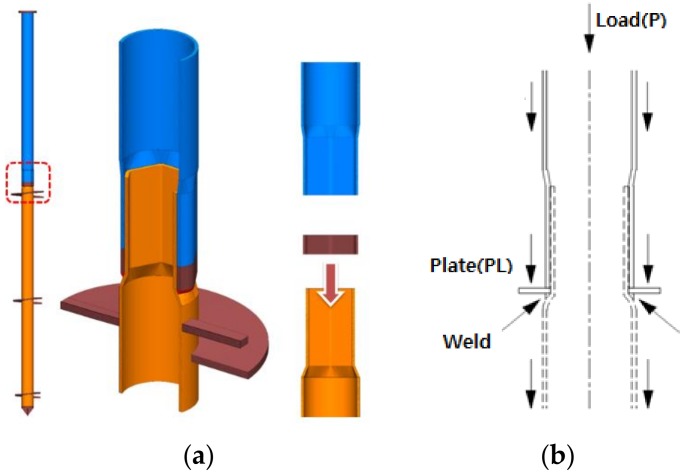
Designing hexagonal joint: (**a**) Hexagonal joint model; (**b**) Concept of load transfer.

Figure 2a shows a hexagon joint model, and Figure 2b shows the concept of load transfer in which load transfer moves to the inner pile through the outer pile when the top load is applied. Where there is no gravity grout plate (PL), the outer pile goes through the curves of the inner pile to generate plastic displacement. Therefore, the gravity grout PL was made to support the movement and conduct gravity grouting.

**Figure 3:**
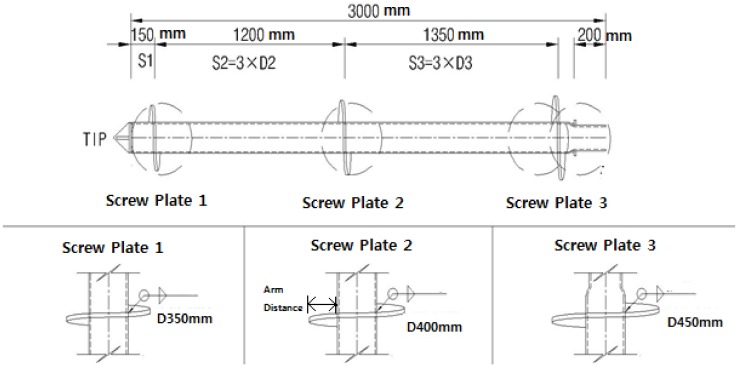
End of the helical pile shown in detail.

In this study, the helix spacing was determined to be 3.0D_h_, as shown in Figure 3, designed according to the individual bearing method through a field ground survey to determine the design load of 600.0 kN. Although the design load of 600.0 kN is supported by the end bearing capacity and the skin friction capacity, the ratio of the end bearing capacity to the bearing capacity of the skin friction capacity is different depending on ground conditions. 

While greater helix plate pitches achieve even deeper rotary penetration, the rolling resistance is greater in the ground. Therefore, 75 mm (three inches), as suggested by [25], was employed, and the helix plate was installed perpendicular to the hollow shaft. Table 1 illustrates the specifications and design load of the helix plate of the helical piles.

## 3. Pile Installation for the Field Test and Results

### 3.1. Planning Pile Installation for the Field Test

**Figure 4:**
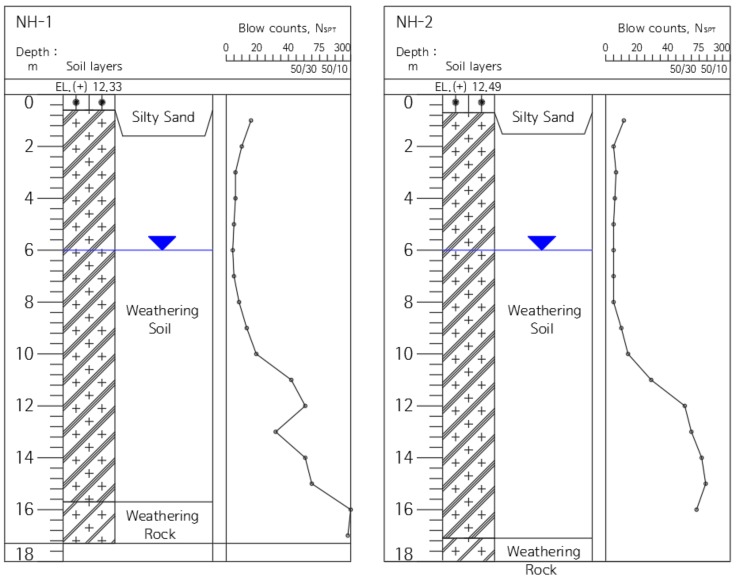
Sectional view of the Site-1 stratum.

**Figure 5:**
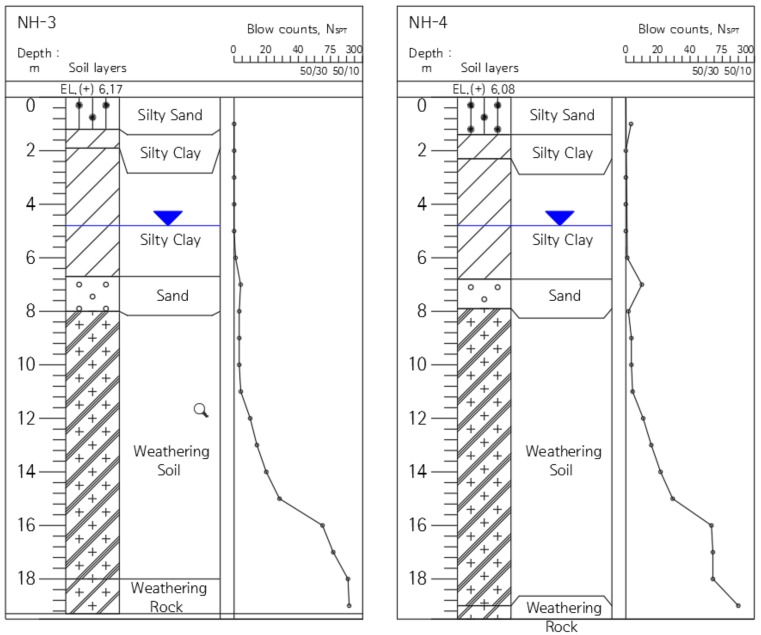
Sectional view of the Site-2 stratum.

The pile installation for the field test was conducted in the local road construction sites of Site-1 (NH-1, 2) shown in Figure 4, and Site-2 (NH-3, 4) shown in Figure 5. The geological features of Site-1 and Site-2 are typical of Korea ground, consisting of sedimentary soil (reclaimed soil), alluvial beds, weathered rock soil, and weathered rock. The sites were selected as areas for piling for the field test.

Fifteen piles were installed in the sites for the field test, respectively, in order to examine the bearing capacity behavior depending on the pile type and grout, and the water/cement (W/C) proportion in the grouting was planned to be 80%, and a compressive, dynamic pile load test was conducted to examine the bearing capacity behavior.

**Figure 6:**
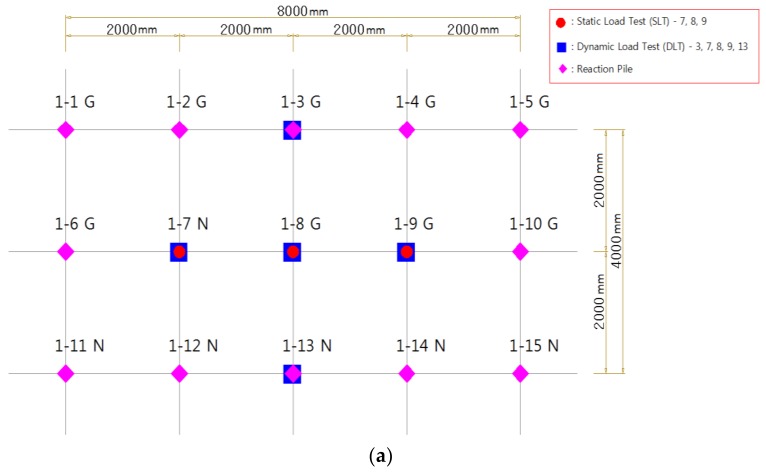

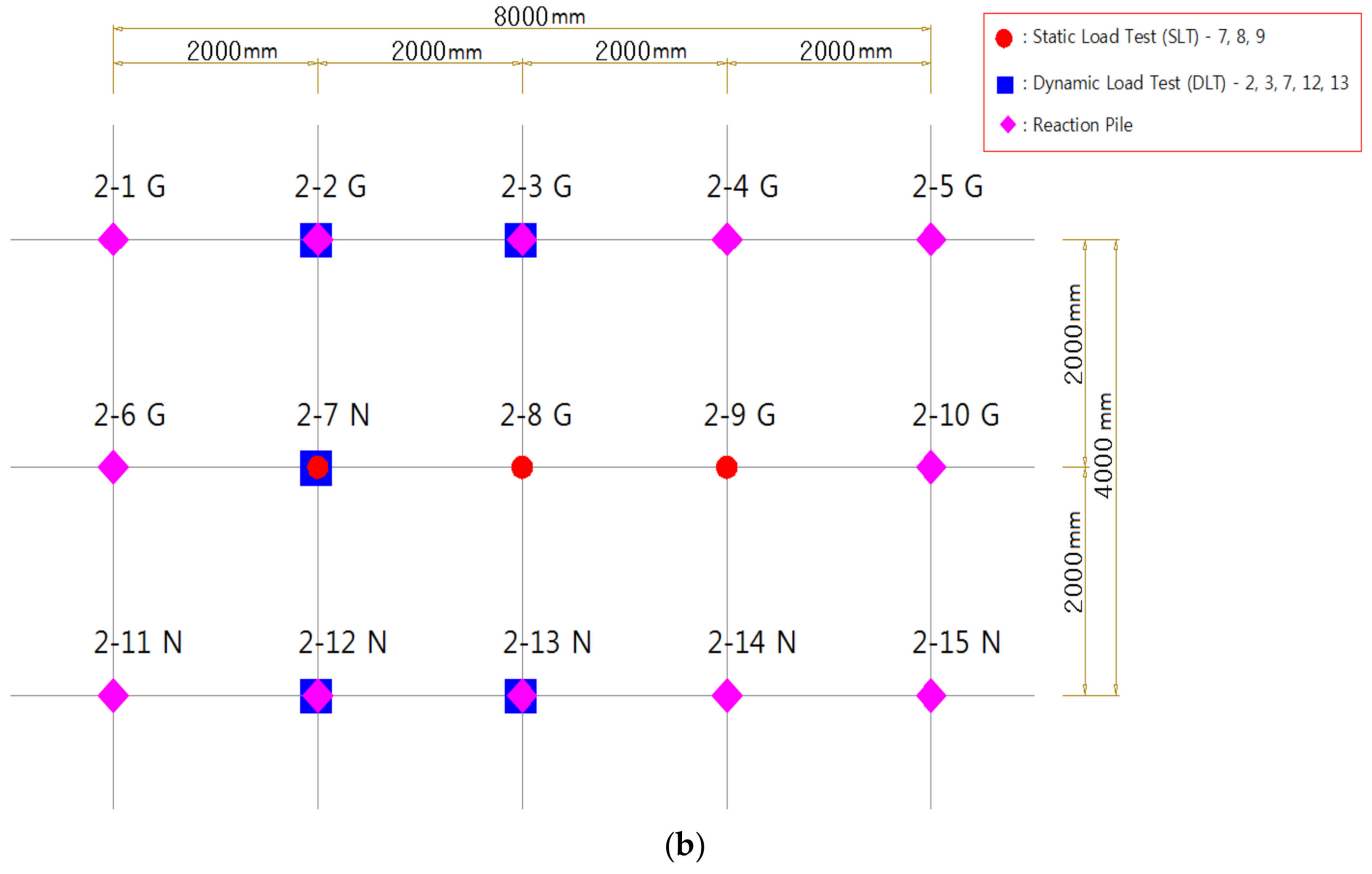
Placing pile for the field pile installation test: (**a**) Site-1 (15 piles); (**b**) Site-2 (15 piles).

Figure 6 shows the pile load test layout. Fifteen piles (3 × 5) were placed in the test sites, respectively, the pile spacing was 2 m, and piles of static load test were installed in the middle column to use the pull-out piles as a reaction pile in the static load test. 

### 3.2. Results of the Static Pile Load Test

Each country has its own standard for the safety factors applied to evaluating the allowable bearing capacity using the static load test, and different standards should be applied depending on the pile types and installation methods. The design bearing capacity of the helical pile used in this study was 600.0 kN, which was calculated using the individual bearing method through subsurface exploration. Therefore, the bearing capacity according to the different regulations applied to evaluating the allowable bearing capacity was compared with the bearing capacity found using conventional methods to consider the characteristics of the helical pile. 

The equation of the individual bearing method can be written as:

(1) $$P_{u}=\underset{n}{\sum }q_{ult}A_{n}+\alpha H\left(\pi D\right)$$

in which q_ult_: Individual end bearing capacity (kN/mm^2^); *A_n_*: Area of helix plate (mm^2^); α: Cohesion between soil and hollow space after (kN/mm^2^); *H*: Shaft length experiencing cylindrical surface friction (mm); *D*: Pile diameter (mm).

Since the helical pile can be constructed to an N-value 30, the depth can be estimated through subsurface exploration. Therefore, the design-bearing capacity can be predicted as in Equation (1).

**Figure 7:**
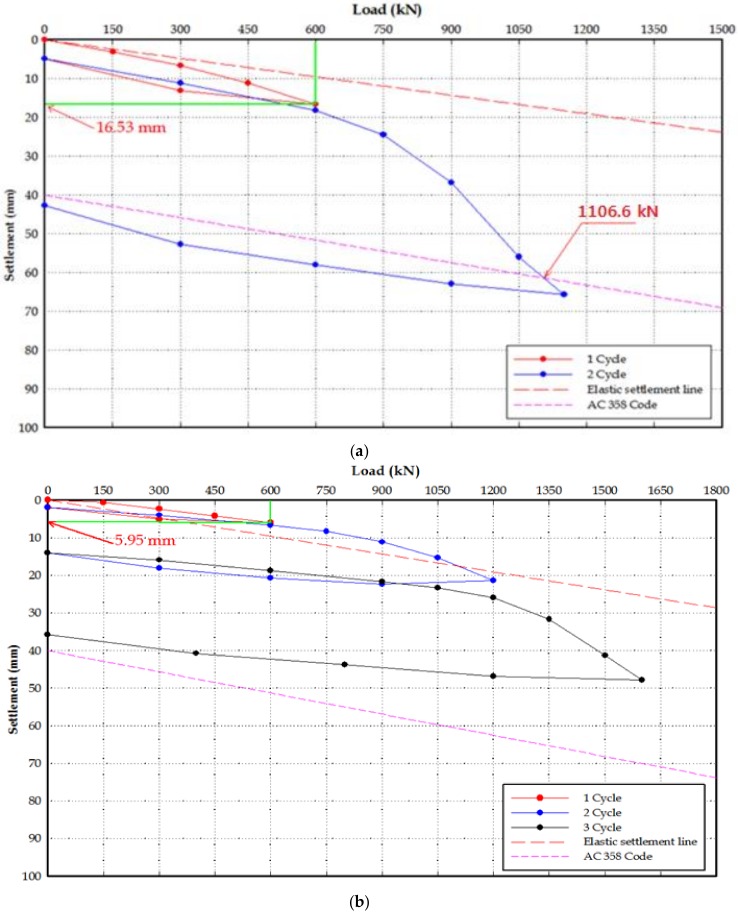

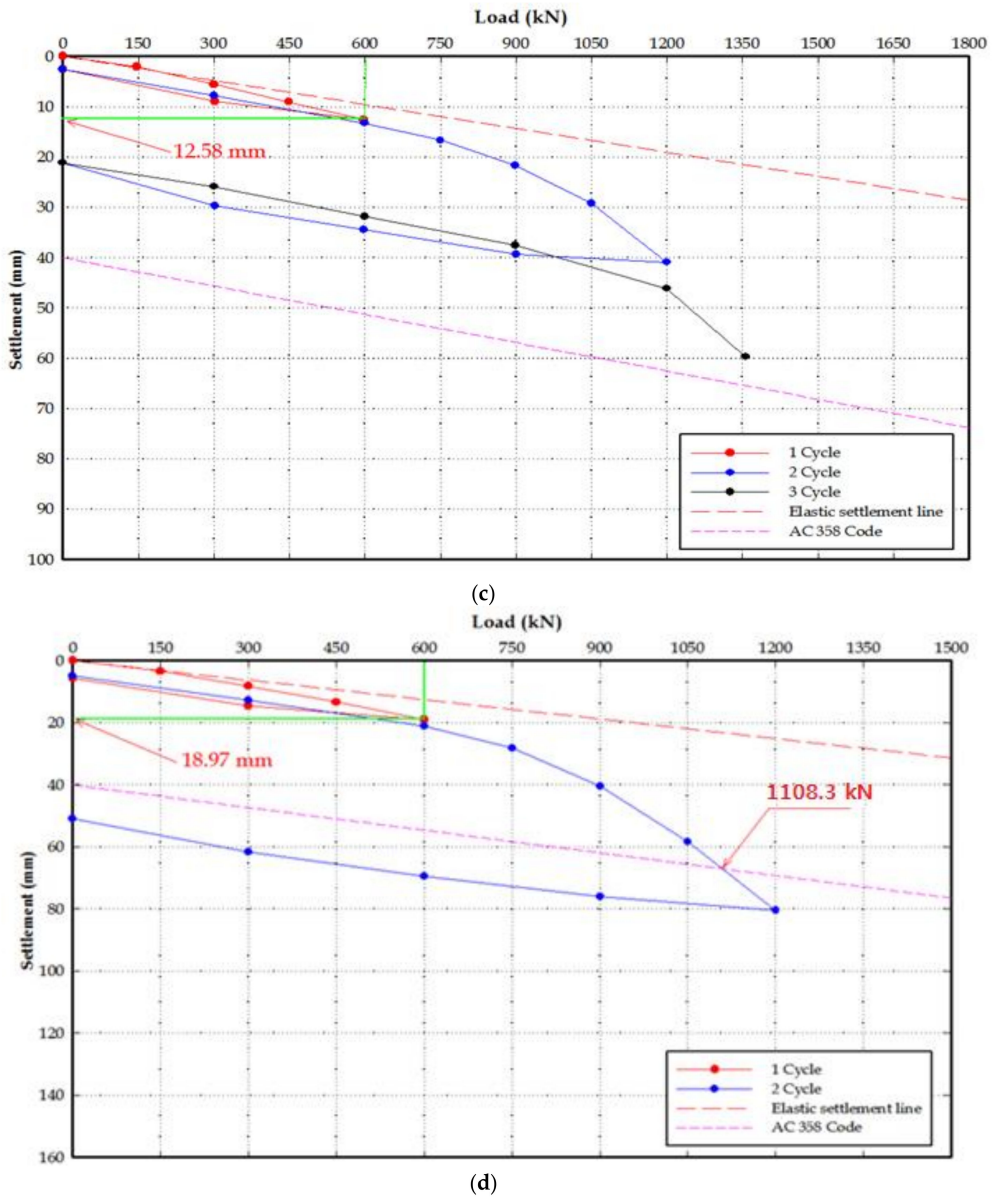

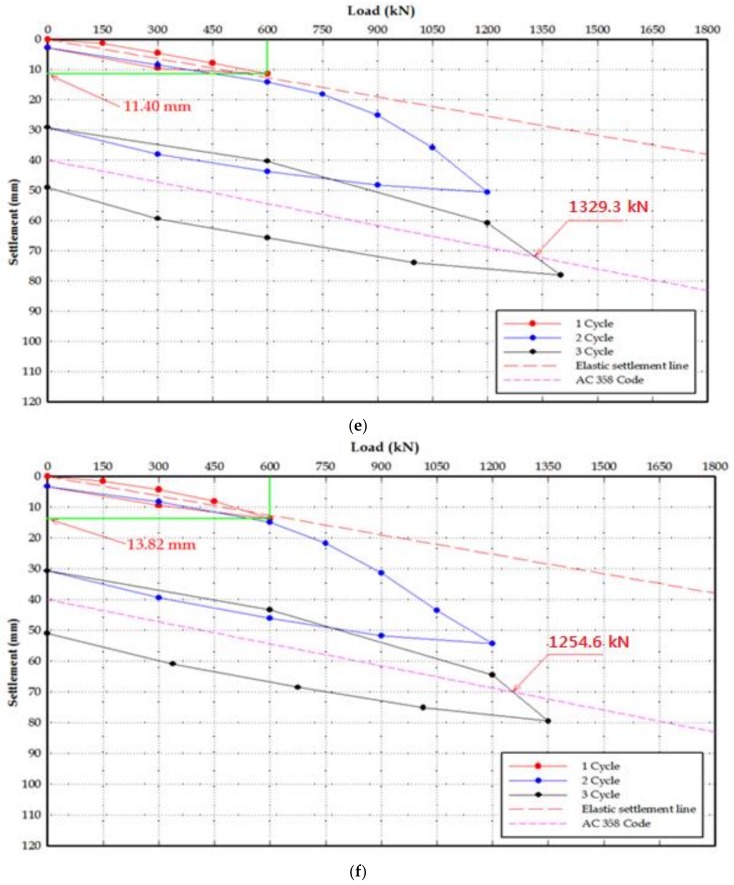
Static load test result for each site: (**a**) Site–1 #7; (**b**) Site–1 #8; (**c**) Site–1 #9; (**d**) Site–2 #7; (**e**) Site–2 #8; (**f**) Site–2 #9.

Figure 7 shows the bearing capacity evaluation according to the helix plate standard using Davisson’s method [26]. Prior studies in other countries show bearing capacity values closest to the 600.0 kN designed using the individual bearing method while applying the AC 358 code. Therefore, for the load test in this study, the AC 358 code was applied to evaluate the bearing capacity.

The static load test was conducted using the static load test method using the pull-out resistance of the surrounding piles; the load that was three times the design load was determined as the maximum test load to conduct the quick maintained load test [27] in order to determine the ultimate load. 

The static load test conducted for the Site-1 and Site-2 test piles showed that the allowable bearing capacity of the gravity grout piles was 678.6–800 kN/pile in Site-1, and 627.3–664.7 kN/pile at Site-2. In the non-grout piles, the allowable bearing capacity was 553.3 kN/pile and 554.2 kN/pile at Sites-1 and 2, respectively, suggesting that gravity grout is required when installing helical piles. The gravity grout piles showed one inch (25.4 mm) which is an allowable settlement standard with a design load of 600.0 kN, suggesting stable settlement.

In particular, pile 1-#8, which was considered installed in weathered rock, experienced a settlement not greater than the allowable settlement (25.4 mm) although the given load was twice the design load, suggesting that an even greater allowable bearing capacity can be applied where the helical pile is installed in the weathered rock. Therefore, it is necessary to further study how to establish a method for installing the helical piles in weathered rocks to achieve even greater design loads.

Table 2 provides the results of static load test for each site.

### 3.3. Analysis of the Dynamic Pile Load Test Results

Table 3 illustrates the results of the CAPWAP (case pile wave analysis program) [28]. 

Dynamic pile load tests are the end of the initial driving test and restrike test. The end of the initial driving test is performed to confirm the performance of the construction equipment and determine the integrity test. The restrike test is performed after a certain period of time after construction to confirm the effect with time on the pile.

The restrike method was used for the respective tested piles 28 days after construction, and a drop hammer with a ram weight of 23.0 kN was used in the dynamic pile load test to minimize the number of test strikes and thus minimize ground disturbance in the test. 

The dynamic pile load test for the piles in Site-1 shows the allowable bearing capacity was 625.0–817.9 kN/pile for the gravity grout piles, and the allowable bearing capacity was 503.4–619.7 kN/pile for the non-grout piles, suggesting that gravity grouting is required when installing helical piles. 

The dynamic pile load test for the piles in Site-2 shows that the allowable bearing capacity was 620.7–674.6 kN/pile for the gravity grout piles, and the allowable bearing capacity was 426.8–563.9 kN/pile for the non-grout piles, suggesting that gravity grouting is required when installing helical piles.

## 4. Suggested Equations for Calculating the Bearing Capacity 

### 4.1. Suggested Equation for Calculating the Bearing Capacity According to the Load Test Result 

The helical plate and the pile tip penetrated at a depth higher than the weathered soil (N > 50) planned for the test installation in this study, and the piles penetrated even into the weathered rock top in the differential weathered layer of Site-1 (#1–8). Moreover, the presence of gravity grout is known to classify the equation for calculating bearing capacity depending on skin friction capacity changes, and it is necessary to use an appropriate calculation equation after examining the soil layering in the pile installation site to classify the end support layer.

#### 4.1.1. Analysis of the Skin Friction and End Bearing Capacity According to the Load Test Result 

The load transfer test is required to examine the bearing capacity at which skin friction and end bearing capacity is classified to determine the equation for calculating the bearing capacity. However, because the small-diameter steel pipes of the helical piles are installed through rotary penetration, it is impossible to install the lead line of the strain gauge attached to the steel pipes for the load transfer test, and the load transfer test was thus not conducted in this study.

Therefore, with the ultimate bearing capacity of the static load test as a true value in this study, it was planned to separate the skin friction capacity from the end bearing capacity in consideration of the ratio of the cylindrical surface to the end bearing capacity in the dynamic pile load test conducted for the same piles. Therefore, the used data are for the four pile samples of #7, #8 and #9 at Site-1 and #7 at Site-2.

Table 4 illustrates the ultimate bearing capacity according to the static load test results, and Table 5 illustrates the ratio of skin friction to end bearing capacity according to the dynamic pile load test result. Table 6 illustrates the skin friction and end bearing capacity according to the results illustrated in Table 4 and Table 5. The skin friction according to the dynamic pile load test result is just for the shaft section, and the bearing capacity by the pile tip and the helix plate is divided by means of the end bearing capacity.

#### 4.1.2. Analysis of the Design Standard 

In other countries, the calculation equation suggested by [3] is the only equation known for calculating the empirical bearing capacity according to the analysis results of load test data for helical pile installation, and Table 7 illustrates the equation.

In Korea, the method for calculating the end bearing capacity and skin friction capacity depends on the installation method of the pile foundations, in accordance with the applied standards of [30,31].

The aforementioned design standards classify piles into: (1) Driven pile, (2) precast pile, and (3) drilled shafts depending on the method of installation. In particular, conventional pile materials of prestressed high-strength concrete (PHC) piles and steel pipe piles are used to manufacture the driven and precast piles, and the materials used are determined in consideration of characteristics of the top load. In particular, the PHC piles are generally used where the top load is a vertical load, and steel pipe piles are used where the top load has vertical forces, moment and horizontal forces at the same time. 

Therefore, for the conventional piles of PHC and steel pipes, weathered rocks or rock beds are selected as a support layer because it is necessary to select the method of installation and the support layer to maximize the use of the allowable strength of the piles in consideration of the load characteristics to ensure cost effectiveness.

Table 8 illustrates the characteristics depending on the installation method and the equations for calculating the bearing capacity.

#### 4.1.3. Application of the Design Standard for Establishing the Equation for Calculating Bearing Capacity 

The equation for calculating the bearing capacity of helical piles was established after examining the appropriateness of conventional equations by comparing end bearing capacity and skin friction according to the results of the dynamic load test and the static load test with the ultimate bearing capacity calculated using the calculation equation found in [3] (Ⓐ) and the equations Ⓓ and Ⓔ based on Korean design standards.

Conventional equation Ⓐ: The base rock, which is as a support layer is different but uses the same installation method. Equations Ⓓ and Ⓔ: The installation method is different, but the method for calculating the bearing capacity for the same support layer (base rock) is applied to the analysis and comparison to establish an equation for calculating bearing capacity.

Table 9 illustrates the comparison of the skin friction and end bearing capacity according to the load test with the end and skin friction calculated using the conventional equations. The experimental equations of Table 10 are proposed according to the ground condition (weathered soil or rock) and grout condition (grout or non-grout).

Piles 1–8 supported with the weathered rock in the differential weathered layer were expected to have greater end bearing capacity. In consideration of this, the calculation equation was determined. 

For the skin friction in the bearing capacity equations for the precast pile, the coefficient was 2.0–2.5. However, based on the experimental equation of this study, it was confirmed that the coefficient of 1.0 was suitable because the skin friction at the non-grout of helical pile was smaller than the skin friction of the precast pile.

### 4.2. Equation for Calculating the Bearing Capacity Considering the Relation between Torque (T) and Ultimate Bearing Capacity 

#### 4.2.1. Data Analysis 

The analysis of the relation between torque (T) and the ultimate bearing capacity aimed to enhance the reliability of T-qu relation by analyzing torque (T) and the ultimate bearing capacity (qu) of the piles that underwent the static load test. 

**Figure 8:**
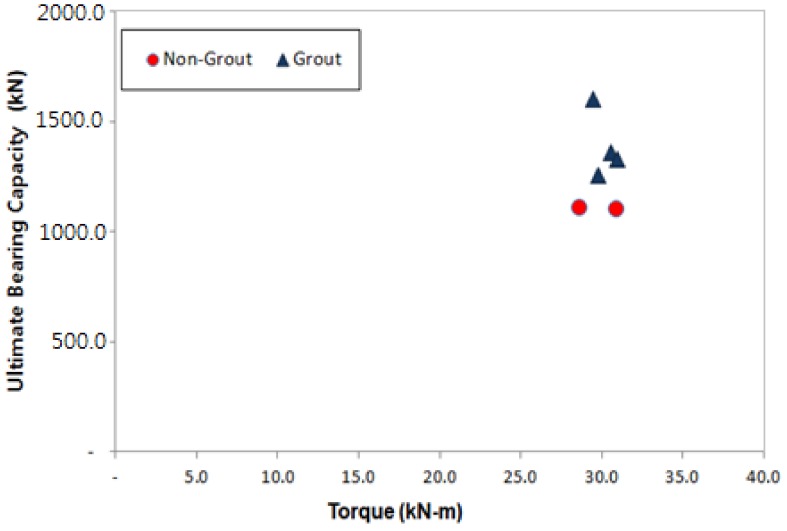
Torque (T)-ultimate bearing capacity (qu).

The following were applied to this study: STK490 (Posco, Gwangyang, Korea), the selection of steel pile of Ø165.2–7.5 t, helix plate specifications of Ø350–450 mm and gravity grout. The aforementioned materials were used to ensure the design load of 600.0 kN, and Table 11 and Figure 8 show the measured final torques and ultimate bearing capacity.

#### 4.2.2. Bearing Capacity Coefficient (kt) According to the Analysis of the Relation between Torque (T)–Ultimate Bearing Capacity (qu) 

For the analysis of the relation between torques (T)–ultimate bearing capacity (qu), the piles were divided into non-grout piles and grout piles to determine the bearing capacity coefficient (kt).

The analysis shows that the vertical bearing capacity quality control of all piles was implemented and the reliability of the vertical bearing capacity could be enhanced for the empirical bearing capacity coefficient kt because the vertical bearing capacity of the piles installed in the sites was known. In addition, the bearing capacity coefficient (kt) obtained from the non-grout piles and the grout piles can be used as illustrated in Equations (2) and (3).

qut (kN) = T × kt(2)

qat (kN) = qut/Fs(3)

in which qut: Ultimate vertical bearing capacity (kN) by kt; qat: Allowable vertical bearing capacity (kN) by kt; T: Final torque (kN·m) measured during installation; kt: Bearing capacity coefficient(m^−1^); and Fs: Safety factor (=2, based on AC358).

**Figure 9:**
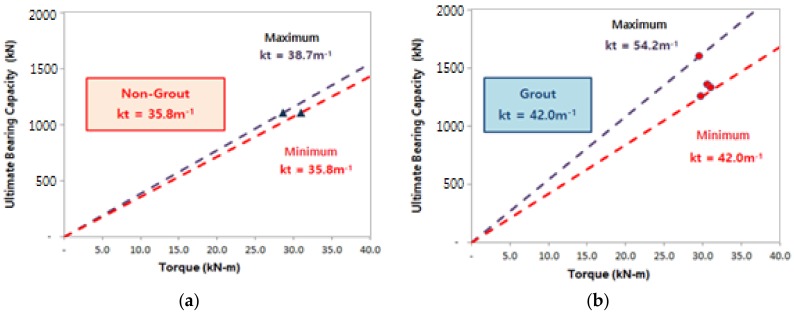
Results of the analysis of the relation between torque (T)–ultimate bearing capacity (qu): (**a**) Non-grout pile; (**b**) Grout pile.

Table 12 and Table 13 and Figure 9 show the results of the analysis of relation between torque (T)–ultimate bearing capacity (qu) for the non-grout piles and grout piles. 

## 5. Summary and Conclusions

This study aimed to improve the hollow shaft model, making it a hexagonal joint that does not require welding or bolts in compressive loading. The following conclusions were drawn by analyzing the static load test results from the field test, dynamic pile load test, and comparing the bearing capacity estimated with the measured torque for Korean ground.

(1) The analysis results of the static load test through the field pile load test shows that the allowable bearing capacity of the gravity grout piles was 678.6–800 kN/pile in Site-1 and 627.3–664.7 kN/pile in Site-2. The allowable bearing capacity of the non-grout piles was smaller than the design load of 600.0 kN/pile in both Sites-1 and 2, suggesting that gravity grouting is required to install helical piles. Moreover, the design load of 600.0 kN resulted in one inch (25.4 mm), which is the allowable settlement standard, suggesting stable settlement.
(2) The analysis of the dynamic pile load test through the field pile load test shows that the allowable bearing capacity of the gravity grout piles was 625.0–817.9 kN/pile in Site-1 and 620.7–674.6 kN/pile in Site-2. The allowable bearing capacity of the non-gravity-grout piles was smaller than 600.0 kN/pile at Sites-1 and 2, similar to the static load test results.
(3) The result of the equation for calculating the empirical bearing capacity in consideration of the load test result, the end bearing capacity (kN) of weathered soil support + non-grout and grout piles was q_p_ = 100 × N × Ap, and the end bearing capacity(kN) of the weathered rock support + grout piles was q_p_ = 150 × N × Ap. Moreover, the skin friction capacity (kN) of the weathered soil support + non-grout piles was q_s_ = 1.0 × N × As, and the skin friction capacity (kN) of the weathered soil support and weathered rock support + grout piles was q_s_ = 5.0 × N × As. The equation for calculating the empirical bearing capacity was established with a small number of samples, and can be used as basic data.
(4) For the equation for calculating the bearing capacity in consideration of torque (T) during pile installation, the bearing capacity coefficient (kt) of non-grout piles was 35.8 m^−1^, allowing for quality control. The bearing capacity coefficient (kt) of the weathered soil support + grout piles was 42.0 m^−1^, and the bearing capacity coefficient (kt) of the weathered rock support piles was 54.2 m^−1^, allowing for quality control. The equation for calculating the bearing capacity in consideration of torque (T) was established with a small number of samples, as for the empirical bearing capacity, and can be used as basic data.

## Figures and Tables

**Table 1:** Specifications and design load of the helix plate.

Category	Plate Diameter (D_h_)	Steel Pipe Diameter (d)	Net Sectional Area of Plate	Yield Strength (MPa)	Design Load(kN)	Arm Distance (mm)
D1	350 mm	165.2 mm	0.075 m^2^	315	142	92.4
D2	400 mm		0.104 m^2^	315	197	117.4
D3	450 mm		0.138 m^2^	315	261	142.4

**Table 2:** Results of the static load test for each site.

Site	Test Location	Penetration Depth (m)	Max. Test Load (kN)	Total Settlement (mm)	Net Settlement(mm)	UltimateLoad (kN)	Allowable Bearing Capacity (kN/pile)			Grout
							Safety Factor (AC 358)	Allowable Bearing Capacity	Design Load	
Site-1	#7	12.10	1150.0	65.73	42.67	1106.6	2.0	553.3	600.0	×
	#8	12.10	1600.0	47.81	35.71	1600.0	2.0	800.0	600.0	100%
	#9	12.00	1356.0	59.67	-	1356.0	2.0	678.0	600.0	80%
Site-2	#7	15.90	1200.0	80.51	50.88	1108.3	2.0	554.2	600.0	×
	#8	16.10	1400.0	78.00	49.06	1329.3	2.0	664.7	600.0	80%
	#9	16.05	1350.0	79.51	50.97	1254.6	2.0	627.3	600.0	80%

**Table 3:** Results of the dynamic pile load test for each site.

Site	Pile No.	Test	Allowable Bearing Capacity (kN/pile)				Grout
			Perko(F.S = 1.5)	A.P.C(F.S = 1.8)	Application(Min.)	Design Load	
Site-1	#3	R	810.5	675.4	675.4	600.0	O
	#7	R	604.1	503.4	503.4	600.0	×
	#8	R	981.5	817.9	817.9	600.0	O
	#9	R	750.0	625.0	625.0	600.0	O
	#13	R	743.6	619.7	619.7	600.0	×
Site-2	#2	R	744.8	620.7	620.7	600.0	O
	#3	R	809.5	674.6	674.6	600.0	O
	#7	R	512.2	426.8	426.8	600.0	×
	#12	R	657.3	547.7	547.7	600.0	×
	#13	R	676.7	563.9	563.9	600.0	×

Note: Perko: Helical pile design and installation [3]; A.P.C: Australian Piling Code [29].

**Table 4:** Ultimate bearing capacity according to the static load test results.

Pile No.	End Support Layer	Grout	Ultimate Bearing Capacity (kN)
Site-1(#7)	Weathered soil	No	1106.6
Site-1(#8)	Weathered rock	Yes	At least 1600.0
Site-1(#9)	Weathered soil	Yes	1356.0
Site-2(#7)	Weathered soil	No	1108.3

**Table 5:** Ratio of skin friction to end bearing capacity according to dynamic pile load test results.

Pile No.	Allowable Bearing Capacity (kN)		Bearing Capacity Ratio	
	Skin Friction Capacity	End Bearing Capacity	Skin Friction Capacity	End Bearing Capacity
Site-1(#7)	13.1	490.4	2.6%	97.4%
Site-1(#8)	104.1	713.9	12.7%	87.3%
Site-1(#9)	79.7	545.3	12.7%	87.3%
Site-2(#7)	7.4	419.4	1.7%	98.3%

**Table 6:** Classification of skin friction and end bearing capacity with load test results.

Pile No.	End Support Layer	Grout	Ultimate Bearing Capacity (kN)	
			Skin Friction Capacity	End Bearing Capacity
Site-1(#7)	Weathered soil	No	28.8	1077.8
Site-1(#8)	Weathered rock	Yes	203.2	At least 1396.8
Site-1(#9)	Weathered soil	Yes	172.2	1183.8
Site-2(#7)	Weathered soil	No	18.8	1089.5

**Table 7:** Perko’s (2009) equation for calculating bearing capacity.

Classification		Equation for Calculating Bearing Capacity	Remarks
Ⓐ	End bearing capacity	q_p_ = β × λ_SPT_ × An β = bearing capacity coefficient Clay: 11, sand: 12, weathered rock: 13 λ_SPT_ = 6.2 kPa/Blow Count (N) An: Helix plate area(mm^2^)	⇒ Equation for calculating end bearing capacity for each stratum Clay = 682 × N × An Sand = 74.4 × N × An Weathered rock = 80.6 × N × An
	Cylindrical surface Friction	q_s_ = α × H × (π × d) α = skin friction (kN/mm^2^)	H = shaft length experiencing cylindrical surface friction (mm) d = pile diameter(mm)

**Table 8:** Characteristics depending on the installation method and experimental equations for calculating bearing capacity.

Classification	Installation Method	Support Layer	Equation for Calculating Ultimate Bearing Capacity		
Driven pile	Conventional piles are installed by using a drop or hydraulic hammer.	Rock bed equivalent to at least weathered rock	Ⓑ	End	q_p_ = 300 × N × Ap
				Cylindrical surface	q_s_ = 2.0 × N × As
Precast pile	A boring machine is first used to bore the ground and the pile is inserted and then driven.	Rock bed equivalent to at least weathered rock	Ⓒ	end	q_p_ = (200–250) × N × Ap
				Cylindrical surface	q_s_ = (2.0–2.5) × N × As
	A boring machine is first used to mix the end with grout and the pile is inserted.		Ⓓ	End	q_p_ = 150 × N × Ap
				Cylindrical surface	q_s_ = (2.0–2.5) × N × As
Drilled shafts	A boring machine is first used to bore the ground, and the reinforced steel net and concrete are then laid to install the pile.	Earth and sand ~rock bed (earth and sand type is applied)	Ⓔ	End	q_p_ = 100 × N × Ap
				Cylindrical surface	q_s_ = (3.3–5.0) × N × As

Note: N: SPT–number of blow counts; Ap: Average area of helix plate; As: Circumference of semi-hollow shaft.

**Table 9:** Comparison of the end bearing capacity and skin friction capacity.

Classification			Pile No.			
			1–7	1–8	1–9	2–7
Skin friction capacity	Load test (①)		28.8 kN	203.2 kN	172.2 kN	18.8 kN
	Conventional equation	Ⓐ	55.7 kN	99.2 kN	98.0 kN	50.2 kN
		Ⓓ	**28.8 kN**	40.8 kN	40.5 kN	**20.6 kN**
		Ⓔ	144.1 kN	**204.1 kN**	**202.6 kN**	102.9 kN
	Ratio (%)	Ⓐ/①	193.4%	48.8%	56.9%	267.0%
		Ⓓ/①	100.0%	20.1%	23.5%	109.6%
		Ⓔ/①	500.3%	100.4%	117.7%	547.3%
	Result of examination		Similar to result of equation D	Similar to result of equation E	Similar to result of equation E	Similar to result of equation D
End bearing capacity	Load test (①)		1077.8 kN	At least 1,396.8 kN	1183.8 kN	1089.5 kN
	Conventional equation	Ⓐ	869.5 kN	830.8 kN	830.3 kN	874.8 kN
		Ⓓ	1618.2 kN	**1545.3 kN**	1545.3 kN	1628.1 kN
		Ⓔ	**1078.8 kN**	1030.2 kN	**1030.2 kN**	**1085.4 kN**
	Ratio (%)	Ⓐ/①	80.7%	59.4%	70.1%	80.3%
		Ⓓ/①	150.1%	110.6%	130.5%	149.4%
		Ⓔ/①	100.1%	73.8%	87.0%	99.6%
	Result of examination		Similar to result of equation E	Similar to result of equation D	Similar to result of equation E	Similar to result of equation E
Bearing capacity equation reliability			100.1%	109.3%	90.9%	99.8%

**Table 10:** Experimental equations for calculating the bearing capacity of the helical pile.

Classification		End Bearing Capacity (kN)	Skin Friction Capacity (kN)	Remarks
	Support by weathered soil + Non-grout	q_p_ = 100 × N × Ap	q_s_ = 1.0 × N × As	N: SPT–number of blow counts Ap: Average area of helix plate As: Circumference of semi-hollow shaft
	Support by weathered soil + grout		q_s_ = 5.0 × N × As	
	Support by weathered rock + grout	q_p_ = 150 × N × Ap		

**Table 11:** Measured torque (T) and ultimate bearing capacity (qu).

Pile No.	Installation Method	Final Torque (T, kN·m)	Ultimate Bearing Capacity (qu, kN)	Remarks
1–7	Rotary penetration	30.9	1106.6	Non-Grout
1–8	Rotary penetration + Grout	29.5	At least 1600	Increased friction by gravity grout
1–9	Rotary penetration + Grout	30.6	1356.0	
2–7	Rotary penetration	28.6	1108.3	Non-Grout
2–8	Rotary penetration + Grout	31.0	1329.3	Increased friction by gravity grout
2–9	Rotary penetration + Grout	29.8	1254.6	

**Table 12:** T-qu analysis result for non-grout piles.

Pile No.	Torque (T, kN·m)	kt (m^−1^)	Ultimate Bearing Capacity (kN)		Bearing Capacity Ratio (ⓑ/ⓐ)
			Measurement	Calculation	
			Static Load Test Result ⓐ	qut = T × kt ⓑ	
1–7	30.9	35.8	1106.6	1106.2	100%
2–7	28.6	38.7	1108.3	1106.8	99.9%
Analysis result	The analysis of relation between T-qu for the non-grout piles shows that kt = 35.8 m^−1^ is applicable to weathered soil support piles.				

**Table 13:** T-qu analysis result for grout piles.

Pile No.	Torque (T, kN·m)	kt (m^−1^)	Ultimate Bearing Capacity (kN)		Bearing Capacity Ratio (ⓑ/ⓐ)
			Measurement	Calculation	
			Static Load Test Result ⓐ	qut = T × kt ⓑ	
1–8	29.5	54.2	At least 1600	1598.9	99.9%
1–9	30.6	44.3	1356.0	1355.6	99.8%
2–8	31.0	42.8	1329.3	1326.8	99.8%
2–9	29.8	42.0	1254.6	1251.6	99.8%
Analysis result	The analysis of relation T-qu for the grout piles shows that kt = 54.2 m^−1^ is applicable to weathered rock support piles and kt = 42.0 m^−1^ to the weathered soil support piles.				
